# Crystal structure of 3,4-di­chloro­anilinium hydrogen phthalate

**DOI:** 10.1107/S2056989015010300

**Published:** 2015-06-03

**Authors:** Muhammad Shahid, Muhammad Nawaz Tahir, Muhammad Salim, Munawar Ali Munawar

**Affiliations:** a Department of Chemistry, University of the Punjab, Lahore, Punjab, Pakistan; b Department of Physics, University of Sargodha, Sargodha, Punjab, Pakistan

**Keywords:** crystal structure, hydrogen phthalate, hydrogen bonding, π–π stacking inter­actions

## Abstract

In the title salt, C_6_H_6_Cl_2_N^+^·C_8_H_5_O_4_^−^, the carb­oxy­lic acid and carboxyl­ate groups of the anion form dihedral angles of 20.79 (19) and 74.76 (14)°, respectively, with the plane of the benzene ring. In the crystal, mol­ecules are assembled into a two-dimensional polymeric network parallel to (100) *via* N—H⋯O and O—H⋯O hydrogen bonds. In addition, within the layer, there are π–π stacking inter­actions between the benzene rings of the cation and the anion [centroid–centroid distance = 3.6794 (17) Å]. A weak C—H⋯O interaction is also observed.

## Related literature   

For related structures, see: Jagan & Sivakumar (2009 ▸, 2011 ▸); Kozma *et al.* (1994 ▸); Liang *et al.* (2011 ▸); Liu (2012 ▸).
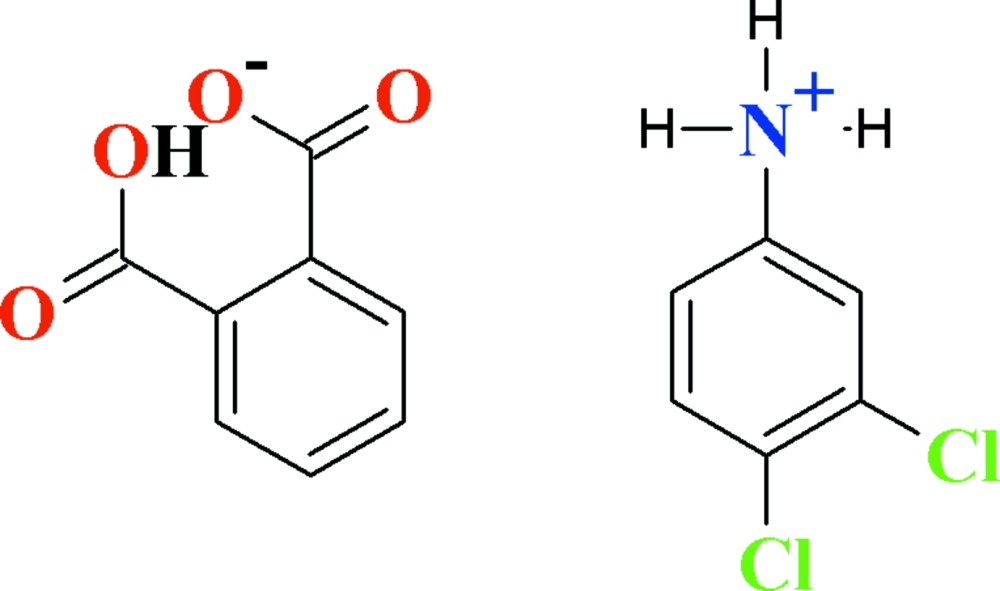


## Experimental   

### Crystal data  

C_6_H_6_Cl_2_N^+^·C_8_H_5_O_4_^−^
*M* *_r_* = 328.14
Monoclinic, 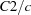

*a* = 29.694 (5) Å
*b* = 7.7536 (13) Å
*c* = 13.125 (2) Å
β = 98.673 (12)°
*V* = 2987.3 (9) Å^3^
*Z* = 8
Mo *K*α radiation
μ = 0.45 mm^−1^
*T* = 296 K
0.34 × 0.28 × 0.16 mm

### Data collection  

Bruker Kappa APEXII CCD diffractometer
Absorption correction: multi-scan (*SADABS*; Bruker, 2005 ▸) *T*_min_ = 0.860, *T*_max_ = 0.935
11652 measured reflections
3248 independent reflections
1849 reflections with *I* > 2σ(*I*)
*R*_int_ = 0.051

### Refinement  

*R*[*F* ^2^ > 2σ(*F* ^2^)] = 0.049
*wR*(*F* ^2^) = 0.133
*S* = 1.02
3248 reflections
192 parameters
H-atom parameters constrained
Δρ_max_ = 0.23 e Å^−3^
Δρ_min_ = −0.26 e Å^−3^

### 

Data collection: *APEX2* (Bruker, 2007 ▸); cell refinement: *SAINT* (Bruker, 2007 ▸); data reduction: *SAINT*; program(s) used to solve structure: *SHELXS97* (Sheldrick, 2008 ▸); program(s) used to refine structure: *SHELXL2014* (Sheldrick, 2015 ▸); molecular graphics: *ORTEP-3 for Windows* (Farrugia, 2012 ▸) and *PLATON* (Spek, 2009 ▸); software used to prepare material for publication: *WinGX* (Farrugia, 2012 ▸) and *PLATON*.

## Supplementary Material

Crystal structure: contains datablock(s) global, I. DOI: 10.1107/S2056989015010300/gk2633sup1.cif

Structure factors: contains datablock(s) I. DOI: 10.1107/S2056989015010300/gk2633Isup2.hkl

Supporting information file. DOI: 10.1107/S2056989015010300/gk2633Isup3.cml

**Fig. 1.**
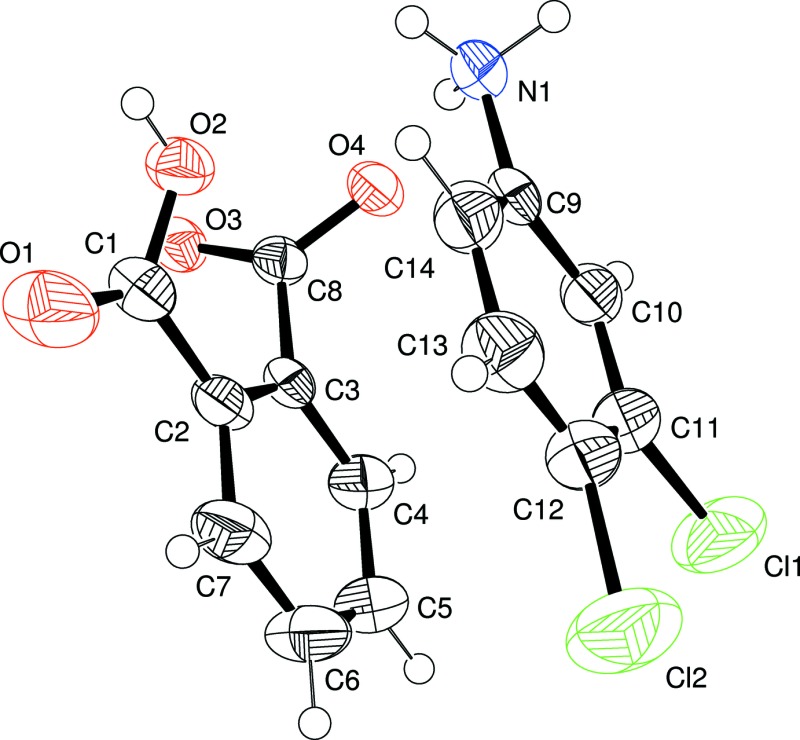
View of the title compound with the atom numbering scheme. The displacement ellipsoids are drawn at the 50% probability level. H-atoms are shown as small circles of arbitrary radii.

. DOI: 10.1107/S2056989015010300/gk2633fig1.tif
View of the title compound with the atom numbering scheme. The displacement ellipsoids are drawn at the 50% probability level. H-atoms are shown as small circles of arbitrary radii.

**Fig. 2.**
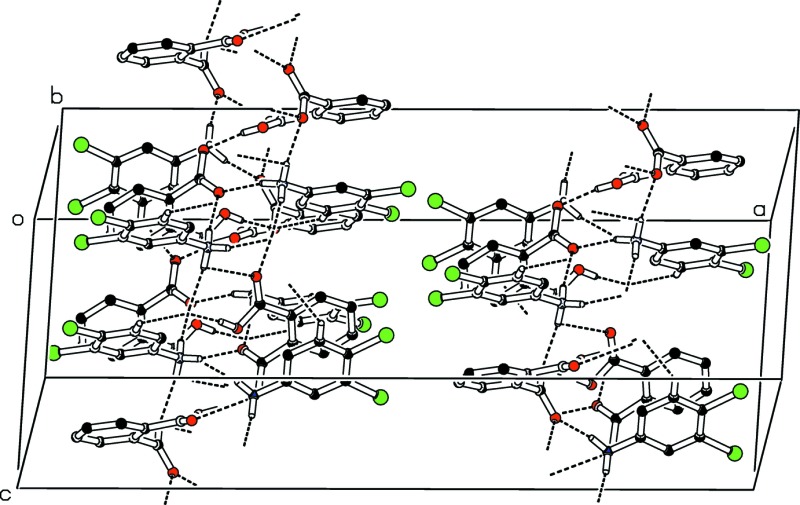
The packing (PLATON; Spek, 2009) of two-dimensional (100) polymeric networks.

PLATON . DOI: 10.1107/S2056989015010300/gk2633fig2.tif
The packing (*PLATON*; Spek, 2009) of two-dimensional (100) polymeric networks.

**Fig. 3.**
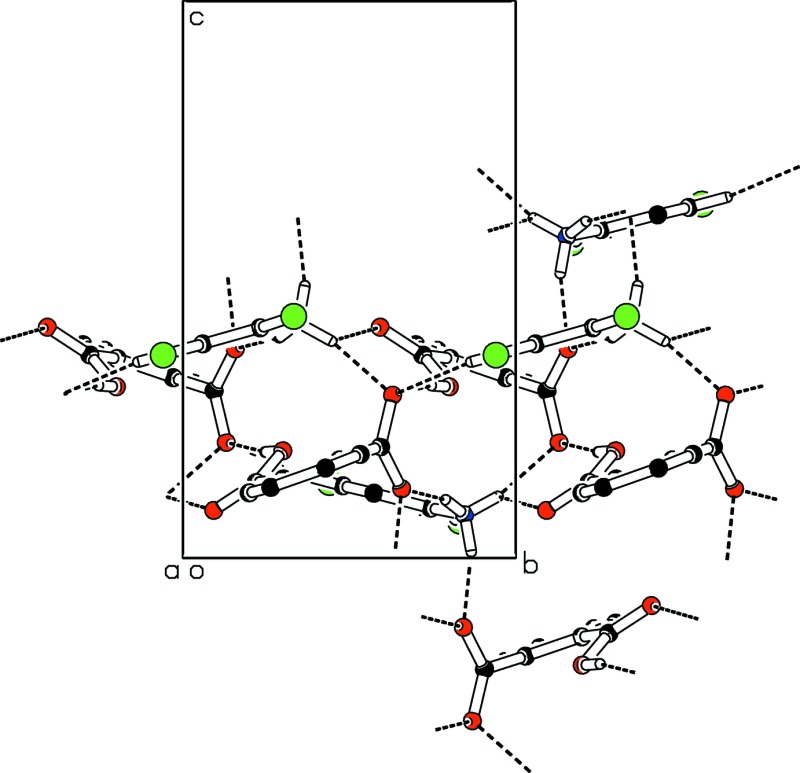
Hydrogen bonds within (100) layer.

. DOI: 10.1107/S2056989015010300/gk2633fig3.tif
Hydrogen bonds within (100) layer.

CCDC reference: 1403731

Additional supporting information:  crystallographic information; 3D view; checkCIF report

## Figures and Tables

**Table 1:** Hydrogen-bond geometry (, )

*D*H*A*	*D*H	H*A*	*D* *A*	*D*H*A*
O2H2O4^i^	0.82	1.77	2.583(2)	171
N1H1*A*O4	0.89	1.98	2.848(3)	164
N1H1*B*O3^ii^	0.89	1.85	2.713(3)	163
N1H1*C*O3^i^	0.89	1.90	2.774(3)	165
C13H13O4^iii^	0.93	2.54	3.328(4)	143

Symmetry codes: (i) 
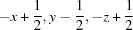
; (ii) 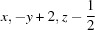; (iii) 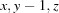.

## supplementary crystallographic information

### S1. Comment

The crystal structures of 4-bromoanilinium hydrogen phthalate (Liang, 2011), (*R*,*S*)-α-phenylethylammonium hydrogen phthalate (Kozma *et al.*, 1994), 4-chloroanilinium hydrogen phthalate (Jagan & Sivakumar, 2009), 3-hydroxyanilinium hydrogen phthalate (Jagan & Sivakumar, 2009), 2-hydroxyanilinium hydrogen phthalate (Jagan & Sivakumar, 2009), 3-Methylanilinium 2-carboxybenzoate (Liu, 2012) and 4-ethoxyanilinium 2- carboxybenzoate (Jagan & Sivakumar, 2011) have been published which are related to the title compound (I, Fig. 1). (I) is synthesized for the study of co-crystallization.

In (I) the benzene ring A (C2—C7) of the phathalate anion is planar with r.m.s. deviation of 0.0024 Å. The carboxylic B (C1/O1/O2) and carboxylate C (C8/O3/O4) groups are oriented at a dihedral angle of 20.79 (19)° and 74.76 (14)°, respectively, with the parent benzene ring A. The molecules form a two dimensional polymeric network parallel to (100) due to N—H···O and O—H···O hydrogen bonds (Table 1, Fig.2). There exist π···π interaction between *Cg1*1···*Cg2*1^i^ [i = *x*, *y*, *z*] with centroid-centroid distance of 3.6794 (17) Å, where *Cg1* and *Cg2* are the centroids of the benzene rings A and E (C9—C14). The topology of two-dimensional hydrogen-bond network in the title compound is the same as in 4-chloroanilinium salt (Jagan & Sivakumar, 2009).

### S2. Experimental

Equimolar quantities of phathalic acid (0.831 g, 5 mmol) and 3,4-dichloroaniline (0.810 g, 5 mmol) were refluxed in 20 ml of methanol for 2 h. The solution was kept at room temperature and colorless plates appeared after two days (m.p. 422–423 K).

### S3. Refinement

The H atoms were positioned geometrically (C–H = 0.93 Å, N–H = 0.89 Å, O—H = 0.82 Å) and refined as riding with *U*_iso_(H) = *xU*_eq_ (C, N, O), where *x* = 1.5 for NH_3_ and hydroxy and *x* =1.2 for aromatic H-atoms.

### Crystal data

**Table:**

C_6_H_6_Cl_2_N^+^·C_8_H_5_O_4_^−^	*F*(000) = 1344
*M**_r_* = 328.14	*D*_x_ = 1.459 Mg m^−^^3^
Monoclinic, *C*2/*c*	Mo *K*α radiation, λ = 0.71073 Å
*a* = 29.694 (5) Å	Cell parameters from 1849 reflections
*b* = 7.7536 (13) Å	θ = 2.8–27.0°
*c* = 13.125 (2) Å	µ = 0.45 mm^−^^1^
β = 98.673 (12)°	*T* = 296 K
*V* = 2987.3 (9) Å^3^	Plate, colorless
*Z* = 8	0.34 × 0.28 × 0.16 mm

### Data collection

**Table:**

Bruker Kappa APEXII CCD diffractometer	3248 independent reflections
Radiation source: fine-focus sealed tube	1849 reflections with *I* > 2σ(*I*)
Graphite monochromator	*R*_int_ = 0.051
Detector resolution: 7.80 pixels mm^-1^	θ_max_ = 27.0°, θ_min_ = 2.8°
ω scans	*h* = −37→37
Absorption correction: multi-scan (*SADABS*; Bruker, 2005)	*k* = −9→9
*T*_min_ = 0.860, *T*_max_ = 0.935	*l* = −15→16
11652 measured reflections	

### Refinement

**Table:**

Refinement on *F*^2^	Primary atom site location: structure-invariant direct methods
Least-squares matrix: full	Secondary atom site location: difference Fourier map
*R*[*F*^2^ > 2σ(*F*^2^)] = 0.049	Hydrogen site location: inferred from neighbouring sites
*wR*(*F*^2^) = 0.133	H-atom parameters constrained
*S* = 1.02	*w* = 1/[σ^2^(*F*_o_^2^) + (0.0503*P*)^2^ + 2.3478*P*] where *P* = (*F*_o_^2^ + 2*F*_c_^2^)/3
3248 reflections	(Δ/σ)_max_ < 0.001
192 parameters	Δρ_max_ = 0.23 e Å^−^^3^
0 restraints	Δρ_min_ = −0.26 e Å^−^^3^

### Special details

**Table:**

Geometry. All e.s.d.'s (except the e.s.d. in the dihedral angle between two l.s. planes) are estimated using the full covariance matrix. The cell e.s.d.'s are taken into account individually in the estimation of e.s.d.'s in distances, angles and torsion angles; correlations between e.s.d.'s in cell parameters are only used when they are defined by crystal symmetry. An approximate (isotropic) treatment of cell e.s.d.'s is used for estimating e.s.d.'s involving l.s. planes.
Refinement. Refinement of *F*^2^ against ALL reflections. The weighted *R*-factor *wR* and goodness of fit *S* are based on *F*^2^, conventional *R*-factors *R* are based on *F*, with *F* set to zero for negative *F*^2^. The threshold expression of *F*^2^ > σ(*F*^2^) is used only for calculating *R*-factors(gt) *etc*. and is not relevant to the choice of reflections for refinement. *R*-factors based on *F*^2^ are statistically about twice as large as those based on *F*, and *R*-factors based on ALL data will be even larger.

### Fractional atomic coordinates and isotropic or equivalent isotropic displacement parameters (Å^2^)

**Table:**

	*x*	*y*	*z*	*U*_iso_*/*U*_eq_	
O1	0.21968 (8)	0.5928 (3)	0.41451 (18)	0.0727 (7)	
O2	0.23502 (6)	0.8002 (2)	0.30759 (13)	0.0427 (5)	
H2	0.2590	0.7471	0.3096	0.064*	
O3	0.21509 (6)	1.1573 (2)	0.37561 (12)	0.0417 (5)	
O4	0.19151 (5)	1.1301 (2)	0.20757 (12)	0.0377 (4)	
C1	0.20865 (9)	0.7204 (4)	0.3646 (2)	0.0416 (7)	
C2	0.16278 (8)	0.8021 (4)	0.35827 (18)	0.0379 (6)	
C3	0.15350 (8)	0.9708 (4)	0.32373 (16)	0.0340 (6)	
C4	0.10931 (9)	1.0324 (4)	0.3140 (2)	0.0476 (7)	
H4	0.1029	1.1443	0.2907	0.057*	
C5	0.07466 (10)	0.9285 (5)	0.3386 (2)	0.0575 (9)	
H5	0.0451	0.9709	0.3316	0.069*	
C6	0.08358 (10)	0.7643 (5)	0.3731 (2)	0.0654 (10)	
H6	0.0601	0.6957	0.3900	0.078*	
C7	0.12732 (10)	0.6995 (4)	0.3830 (2)	0.0533 (8)	
H7	0.1332	0.5872	0.4063	0.064*	
C8	0.18979 (8)	1.0937 (3)	0.30026 (18)	0.0324 (6)	
Cl1	0.03331 (3)	0.83295 (14)	0.06616 (8)	0.0831 (4)	
Cl2	0.04230 (3)	0.44073 (16)	0.13559 (9)	0.0970 (4)	
N1	0.20521 (7)	0.8466 (3)	0.07778 (14)	0.0374 (5)	
H1A	0.2041	0.9466	0.1106	0.056*	
H1B	0.2057	0.8662	0.0111	0.056*	
H1C	0.2303	0.7895	0.1043	0.056*	
C9	0.16540 (8)	0.7446 (4)	0.08969 (16)	0.0334 (6)	
C10	0.12353 (8)	0.8247 (4)	0.07404 (18)	0.0396 (6)	
H10	0.1211	0.9403	0.0551	0.047*	
C11	0.08537 (9)	0.7318 (4)	0.0867 (2)	0.0496 (8)	
C12	0.08933 (9)	0.5597 (5)	0.1155 (2)	0.0530 (8)	
C13	0.13137 (10)	0.4807 (4)	0.1296 (2)	0.0526 (8)	
H13	0.1339	0.3649	0.1480	0.063*	
C14	0.16975 (9)	0.5740 (4)	0.1163 (2)	0.0440 (7)	
H14	0.1982	0.5214	0.1253	0.053*	

### Atomic displacement parameters (Å^2^)

**Table:**

	*U*^11^	*U*^22^	*U*^33^	*U*^12^	*U*^13^	*U*^23^
O1	0.0725 (15)	0.0638 (16)	0.0883 (17)	0.0207 (12)	0.0337 (12)	0.0404 (14)
O2	0.0415 (10)	0.0463 (12)	0.0432 (10)	0.0097 (9)	0.0159 (9)	0.0077 (9)
O3	0.0421 (10)	0.0544 (13)	0.0293 (9)	−0.0072 (9)	0.0077 (8)	−0.0073 (9)
O4	0.0425 (10)	0.0423 (12)	0.0297 (9)	−0.0029 (8)	0.0099 (7)	0.0012 (8)
C1	0.0494 (16)	0.0422 (18)	0.0352 (14)	0.0016 (14)	0.0130 (12)	0.0024 (13)
C2	0.0431 (15)	0.0443 (17)	0.0289 (13)	−0.0027 (13)	0.0134 (11)	0.0022 (12)
C3	0.0350 (13)	0.0466 (18)	0.0215 (12)	−0.0019 (12)	0.0081 (10)	−0.0032 (11)
C4	0.0389 (15)	0.060 (2)	0.0461 (16)	0.0032 (14)	0.0129 (12)	0.0022 (14)
C5	0.0377 (16)	0.079 (3)	0.0589 (19)	−0.0002 (16)	0.0161 (14)	0.0029 (18)
C6	0.0465 (18)	0.087 (3)	0.067 (2)	−0.0160 (19)	0.0239 (15)	0.001 (2)
C7	0.0604 (19)	0.054 (2)	0.0493 (17)	−0.0114 (16)	0.0211 (14)	0.0061 (15)
C8	0.0333 (13)	0.0358 (15)	0.0297 (13)	0.0046 (11)	0.0103 (10)	−0.0041 (11)
Cl1	0.0388 (4)	0.1064 (9)	0.1041 (7)	0.0154 (5)	0.0106 (4)	0.0168 (6)
Cl2	0.0625 (6)	0.1052 (9)	0.1236 (9)	−0.0304 (6)	0.0149 (5)	0.0246 (7)
N1	0.0389 (12)	0.0462 (15)	0.0275 (10)	0.0019 (10)	0.0061 (9)	−0.0017 (10)
C9	0.0376 (14)	0.0429 (17)	0.0205 (11)	−0.0011 (12)	0.0069 (10)	−0.0035 (11)
C10	0.0409 (15)	0.0439 (18)	0.0344 (13)	0.0058 (13)	0.0074 (11)	0.0020 (12)
C11	0.0374 (15)	0.067 (2)	0.0450 (16)	0.0050 (15)	0.0073 (12)	0.0014 (15)
C12	0.0451 (17)	0.065 (2)	0.0493 (17)	−0.0126 (16)	0.0076 (13)	0.0005 (16)
C13	0.060 (2)	0.0458 (19)	0.0521 (17)	−0.0053 (16)	0.0104 (14)	0.0020 (15)
C14	0.0440 (16)	0.0467 (19)	0.0412 (15)	0.0064 (14)	0.0057 (12)	−0.0002 (13)

### Geometric parameters (Å, º)

**Table:**

O1—C1	1.204 (3)	C7—H7	0.9300
O2—C1	1.316 (3)	Cl1—C11	1.718 (3)
O2—H2	0.8200	Cl2—C12	1.726 (3)
O3—C8	1.250 (3)	N1—C9	1.450 (3)
O4—C8	1.257 (3)	N1—H1A	0.8900
C1—C2	1.493 (4)	N1—H1B	0.8900
C2—C7	1.396 (4)	N1—H1C	0.8900
C2—C3	1.398 (4)	C9—C14	1.369 (4)
C3—C4	1.384 (3)	C9—C10	1.377 (3)
C3—C8	1.505 (3)	C10—C11	1.374 (4)
C4—C5	1.383 (4)	C10—H10	0.9300
C4—H4	0.9300	C11—C12	1.388 (5)
C5—C6	1.364 (5)	C12—C13	1.378 (4)
C5—H5	0.9300	C13—C14	1.383 (4)
C6—C7	1.380 (4)	C13—H13	0.9300
C6—H6	0.9300	C14—H14	0.9300
			
C1—O2—H2	109.5	C9—N1—H1A	109.5
O1—C1—O2	124.0 (3)	C9—N1—H1B	109.5
O1—C1—C2	123.3 (2)	H1A—N1—H1B	109.5
O2—C1—C2	112.6 (2)	C9—N1—H1C	109.5
C7—C2—C3	119.3 (3)	H1A—N1—H1C	109.5
C7—C2—C1	117.3 (3)	H1B—N1—H1C	109.5
C3—C2—C1	123.2 (2)	C14—C9—C10	121.5 (2)
C4—C3—C2	119.4 (2)	C14—C9—N1	120.5 (2)
C4—C3—C8	117.4 (3)	C10—C9—N1	118.1 (2)
C2—C3—C8	123.1 (2)	C11—C10—C9	119.2 (3)
C5—C4—C3	120.4 (3)	C11—C10—H10	120.4
C5—C4—H4	119.8	C9—C10—H10	120.4
C3—C4—H4	119.8	C10—C11—C12	120.0 (3)
C6—C5—C4	120.5 (3)	C10—C11—Cl1	118.7 (3)
C6—C5—H5	119.8	C12—C11—Cl1	121.3 (2)
C4—C5—H5	119.8	C13—C12—C11	120.1 (3)
C5—C6—C7	120.3 (3)	C13—C12—Cl2	118.7 (3)
C5—C6—H6	119.9	C11—C12—Cl2	121.2 (2)
C7—C6—H6	119.9	C12—C13—C14	119.9 (3)
C6—C7—C2	120.2 (3)	C12—C13—H13	120.1
C6—C7—H7	119.9	C14—C13—H13	120.1
C2—C7—H7	119.9	C9—C14—C13	119.3 (3)
O3—C8—O4	124.7 (2)	C9—C14—H14	120.3
O3—C8—C3	116.8 (2)	C13—C14—H14	120.3
O4—C8—C3	118.4 (2)		
			
O1—C1—C2—C7	−20.8 (4)	C2—C3—C8—O3	−74.5 (3)
O2—C1—C2—C7	157.5 (2)	C4—C3—C8—O4	−74.4 (3)
O1—C1—C2—C3	162.9 (3)	C2—C3—C8—O4	108.1 (3)
O2—C1—C2—C3	−18.9 (4)	C14—C9—C10—C11	0.8 (4)
C7—C2—C3—C4	−0.5 (4)	N1—C9—C10—C11	−178.8 (2)
C1—C2—C3—C4	175.8 (2)	C9—C10—C11—C12	0.4 (4)
C7—C2—C3—C8	176.9 (2)	C9—C10—C11—Cl1	−179.63 (18)
C1—C2—C3—C8	−6.8 (4)	C10—C11—C12—C13	−1.3 (4)
C2—C3—C4—C5	0.3 (4)	Cl1—C11—C12—C13	178.8 (2)
C8—C3—C4—C5	−177.2 (2)	C10—C11—C12—Cl2	178.2 (2)
C3—C4—C5—C6	0.2 (4)	Cl1—C11—C12—Cl2	−1.8 (4)
C4—C5—C6—C7	−0.5 (5)	C11—C12—C13—C14	0.9 (4)
C5—C6—C7—C2	0.3 (5)	Cl2—C12—C13—C14	−178.6 (2)
C3—C2—C7—C6	0.2 (4)	C10—C9—C14—C13	−1.2 (4)
C1—C2—C7—C6	−176.3 (3)	N1—C9—C14—C13	178.5 (2)
C4—C3—C8—O3	102.9 (3)	C12—C13—C14—C9	0.3 (4)

### Hydrogen-bond geometry (Å, º)

**Table:**

*D*—H···*A*	*D*—H	H···*A*	*D*···*A*	*D*—H···*A*
O2—H2···O4^i^	0.82	1.77	2.583 (2)	171
N1—H1*A*···O4	0.89	1.98	2.848 (3)	164
N1—H1*B*···O3^ii^	0.89	1.85	2.713 (3)	163
N1—H1*C*···O3^i^	0.89	1.90	2.774 (3)	165
C13—H13···O4^iii^	0.93	2.54	3.328 (4)	143

Symmetry codes: (i) −*x*+1/2, *y*−1/2, −*z*+1/2; (ii) *x*, −*y*+2, *z*−1/2; (iii) *x*, *y*−1, *z*.

## References

[bb1] Bruker (2005). *SADABS*. Bruker AXS Inc., Madison, Wisconsin, USA.

[bb2] Bruker (2007). *APEX2* and *SAINT*. Bruker AXS Inc., Madison, Wisconsin, USA.

[bb3] Farrugia, L. J. (2012). *J. Appl. Cryst.* **45**, 849–854.

[bb4] Jagan, R. & Sivakumar, K. (2009). *Acta Cryst.* C**65**, o414–o418. 10.1107/S0108270109025724 19652327

[bb5] Jagan, R. & Sivakumar, K. (2011). *Acta Cryst.* C**67**, o373–o377. 10.1107/S010827011103246X 21979970

[bb6] Kozma, D., Bocskei, Z., Simon, K. & Fogassy, E. (1994). *J. Chem. Soc. Perkin Trans. 2*, pp. 1883–1886.

[bb7] Liang, Z. P. (2011). *Acta Cryst.* E**67**, o1430. 10.1107/S1600536811017727 PMC3120532 21754809

[bb8] Liu, M.-L. (2012). *Acta Cryst.* E**68**, o228. 10.1107/S1600536811054353 PMC3254560 22259509

[bb9] Sheldrick, G. M. (2008). *Acta Cryst.* A**64**, 112–122. 10.1107/S0108767307043930 18156677

[bb10] Sheldrick, G. M. (2015). *Acta Cryst.* C**71**, 3–8.

[bb11] Spek, A. L. (2009). *Acta Cryst.* D**65**, 148–155. 10.1107/S090744490804362X PMC2631630 19171970

