# Supplementary figures and images for: Crystal structure of 3,4-di­chloro­anilinium hydrogen phthalate
Source: Acta Crystallogr E Crystallogr Commun. 2015 Jun 3;71(Pt 7):o446. doi: 10.1107/S2056989015010300 (PMC4518937; doi:10.1107/S2056989015010300)

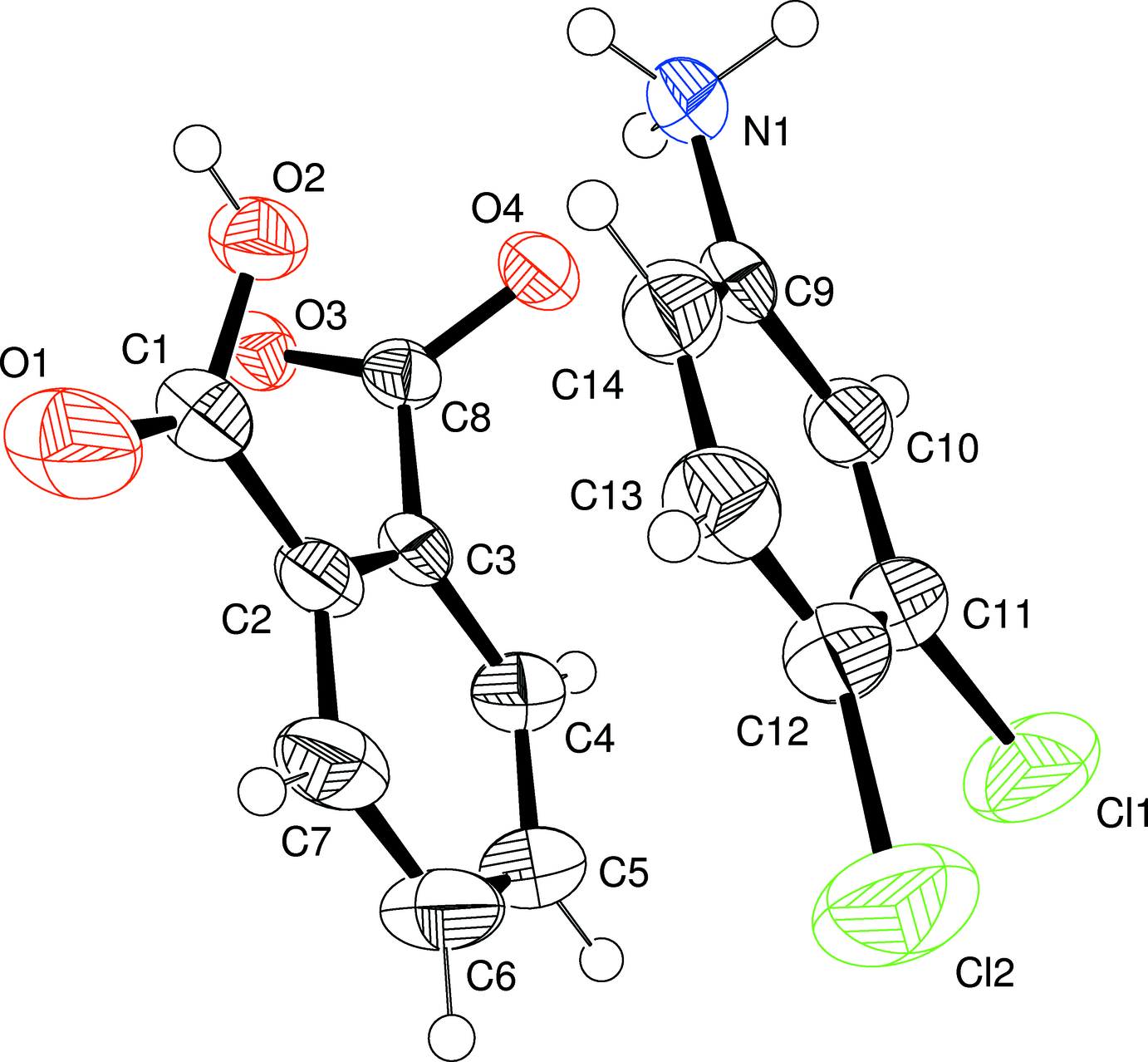

Supplement: Supplementary file 4 [file e-71-0o446-fig1.tif]

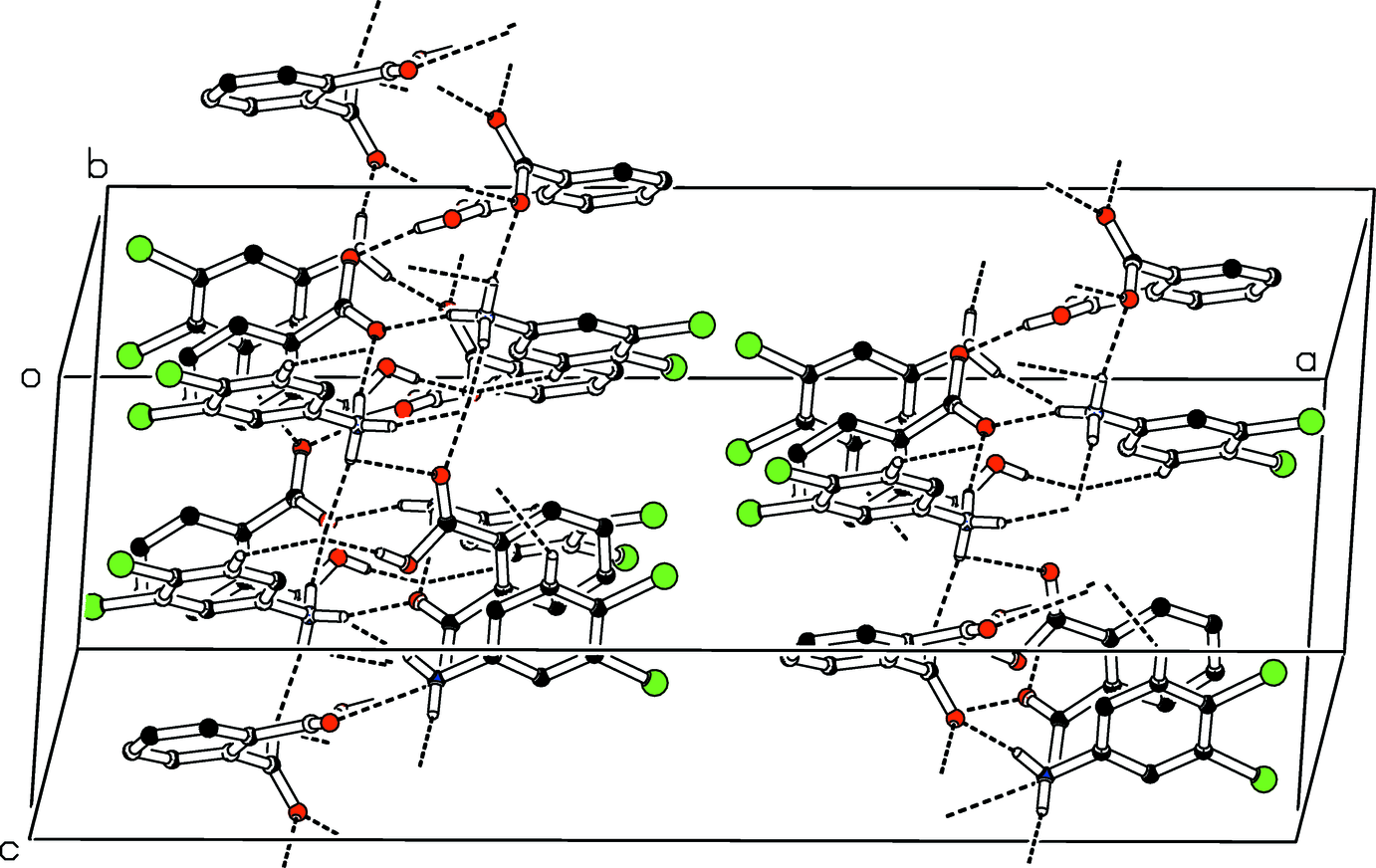

Supplement: Supplementary file 5 [file e-71-0o446-fig2.tif]

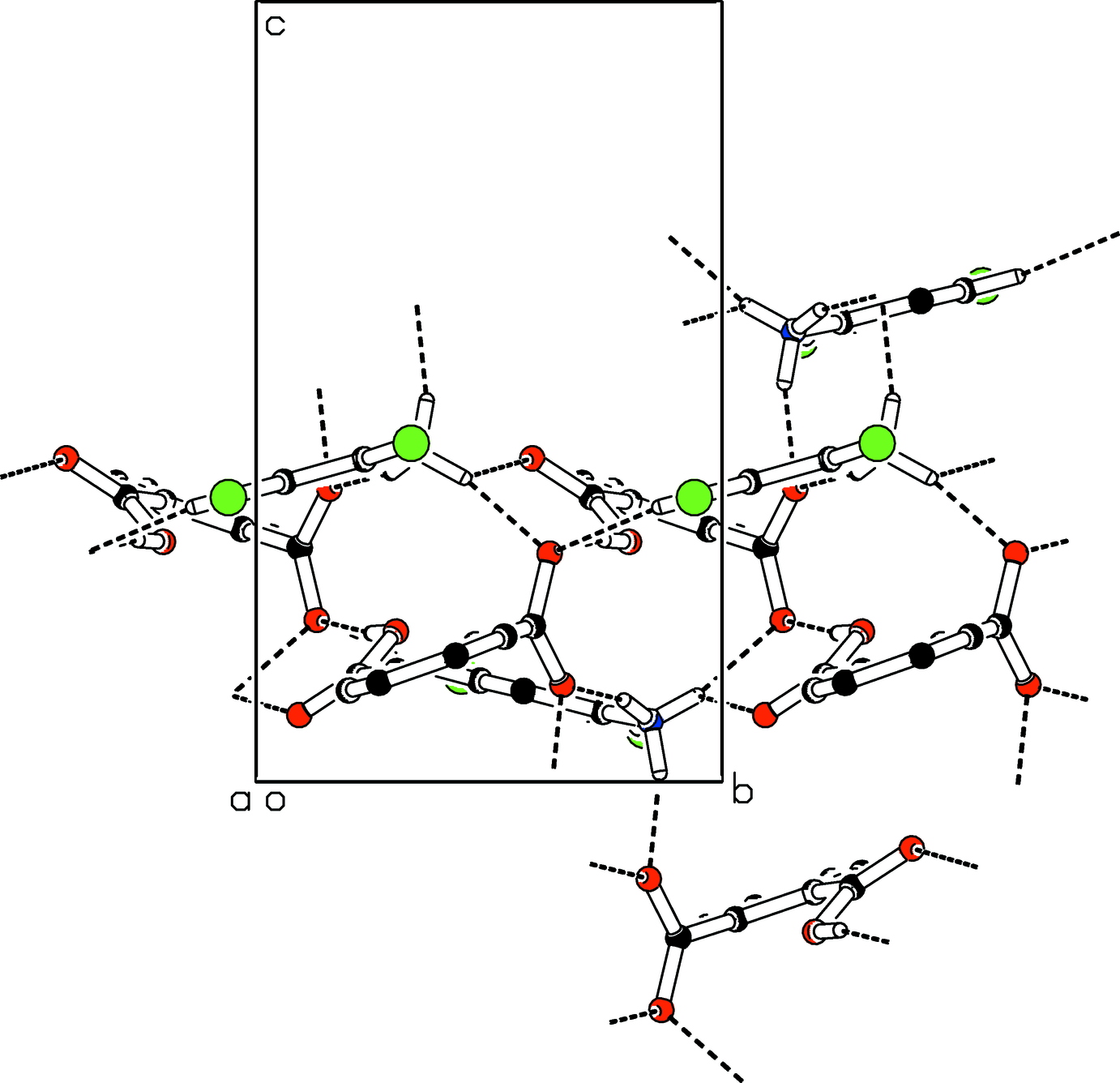

Supplement: Supplementary file 6 [file e-71-0o446-fig3.tif]
